# Immune Control by TRAF6-Mediated Pathways of Epithelial Cells in the EIME (Epithelial Immune Microenvironment)

**DOI:** 10.3389/fimmu.2019.01107

**Published:** 2019-05-16

**Authors:** Teruki Dainichi, Reiko Matsumoto, Alshimaa Mostafa, Kenji Kabashima

**Affiliations:** ^1^Department of Dermatology, Graduate School of Medicine, Kyoto University, Kyoto, Japan; ^2^Department of Dermatology, Beni-Suef University, Beni-Suef, Egypt; ^3^Singapore Immunology Network (SIgN) and Institute of Medical Biology, Agency for Science, Technology and Research (A^*^STAR), Biopolis, Singapore, Singapore

**Keywords:** TRAF6, keratinocyte, EIME, IL-17, NF-kappaB, MAPK

## Abstract

In the protective responses of epithelial tissues, not only immune cells but also non-immune cells directly respond to external agents. Epithelial cells can be involved in the organization of immune responses through two phases. First, the exogenous harmful agents trigger the primary responses of the epithelial cells leading to various types of immune cell activation. Second, cytokines produced by the immune cells that are activated directly by the external agents and indirectly by the epithelial cell products elicit the secondary responses giving rise to further propagation of immune responses. TRAF6 is a ubiquitin E3 ligase, which intermediates between various types of receptors for exogenous agents or endogenous mediators and activation of subsequent transcriptional responses via NF-kappaB and MAPK pathways. TRAF6 ubiquitously participates in many protective responses in immune and non-immune cells. Particularly, epithelial TRAF6 has an essential role in the primary and secondary responses via driving type 17 response in psoriatic inflammation of the skin. Consistently, many psoriasis susceptibility genes encode the TRAF6 signaling players, such as ACT1 (*TRAF3IP2*), A20 (*TNFAIP3*), ABIN1 (*TNIP1*), IL-36Ra (*IL36RN*), IkappaBzeta (*NFKBIZ*), and CARD14. Herein, we describe the principal functions of TRAF6, especially in terms of positive and regulatory immune controls by interaction between immune cells and epithelial cells. In addition, we discuss how TRAF6 in the epithelial cells can organize the differentiation of immune responses and drive inflammatory loops in the epithelial immune microenvironment, which is termed EIME.

## Introduction

The epithelial tissues compose the outermost surface of an organism. Epithelial cells are the first line confronting the exogenous harmful factors, such as toxins and infectious agents. Upon the attack of the offending agents, the epithelial cells not just release their cellular contents, but also respond to each danger by triggering different sets of transcriptional cascades that stimulate a specific type of immune responses. The immune cells that respond directly to the external agents and indirectly to the epithelial cell products are activated and produce a specific set of immune mediators; these in turn activate the epithelial cells again and propagate protective response, which is most effective to solve life-threatening dangers in each situation. Consequently, as well as immune cells, epithelial cells are thought to be involved in the decision and organization of each type of immune responses (1). Therefore, the defect in this shield gives rise to chronic inflammatory skin diseases (1, 2). The mechanistic roles of immune cells and their signaling pathways in the decision of the type of immune responses have been extensively explored. However, the roles of signaling pathways of epithelial cells in the decision of the type of immune responses and their propagation have not been fully understood.

We have demonstrated that tumor necrosis factor (TNF) receptor-associated factor 6 (TRAF6) in the epithelial keratinocytes is essential for driving interleukin (IL)-17-mediated psoriatic inflammation (3). The induction and propagation of type 17 immune responses are fully dependent on the epithelial TRAF6 in the skin of an animal model induced by topical imiquimod. Meanwhile, mice lacking TRAF6 specifically in the gut epithelium show an exacerbation of dextran sulfate sodium (DSS)-induced colitis suggesting a protective role of epithelial TRAF6 in barrier homeostasis and innate protective responses in the gut, which are also mediated by the T helper (T_H_)17 cytokines (4). Taken together, the TRAF6 signaling pathways in the epithelial tissues are expected to play a pivotal role in IL-17-mediated inflammatory and protective responses. Thus, one can speculate that other signaling pathways in epithelial cells are essential in other type of immune responses, and the balance of the dominant cell signaling pathway in epithelial cells may play considerable roles in the decision of immune types. However, the counterpart signaling molecule of TRAF6 in the type 17 immune responses in the epithelial cells in type 1 or 2 immune responses remains obscure.

Here, we describe principal functions of TRAF6 and its roles in immune cells and non-immune epithelial cells. In addition, we provide recent insights into the regulatory mechanisms of the epithelial TRAF6 pathways with the contribution of other ubiquitin E3 ligases, deubiquitinases, and other molecules in type 17 immune responses. Moreover, we propose the function of epithelial TRAF6 in the inflammatory loop of IL-17 through organizing the type 17 epithelial immune microenvironment (EIME).

## Traf6

### Molecular Function of TRAF6

**Figure 1:**
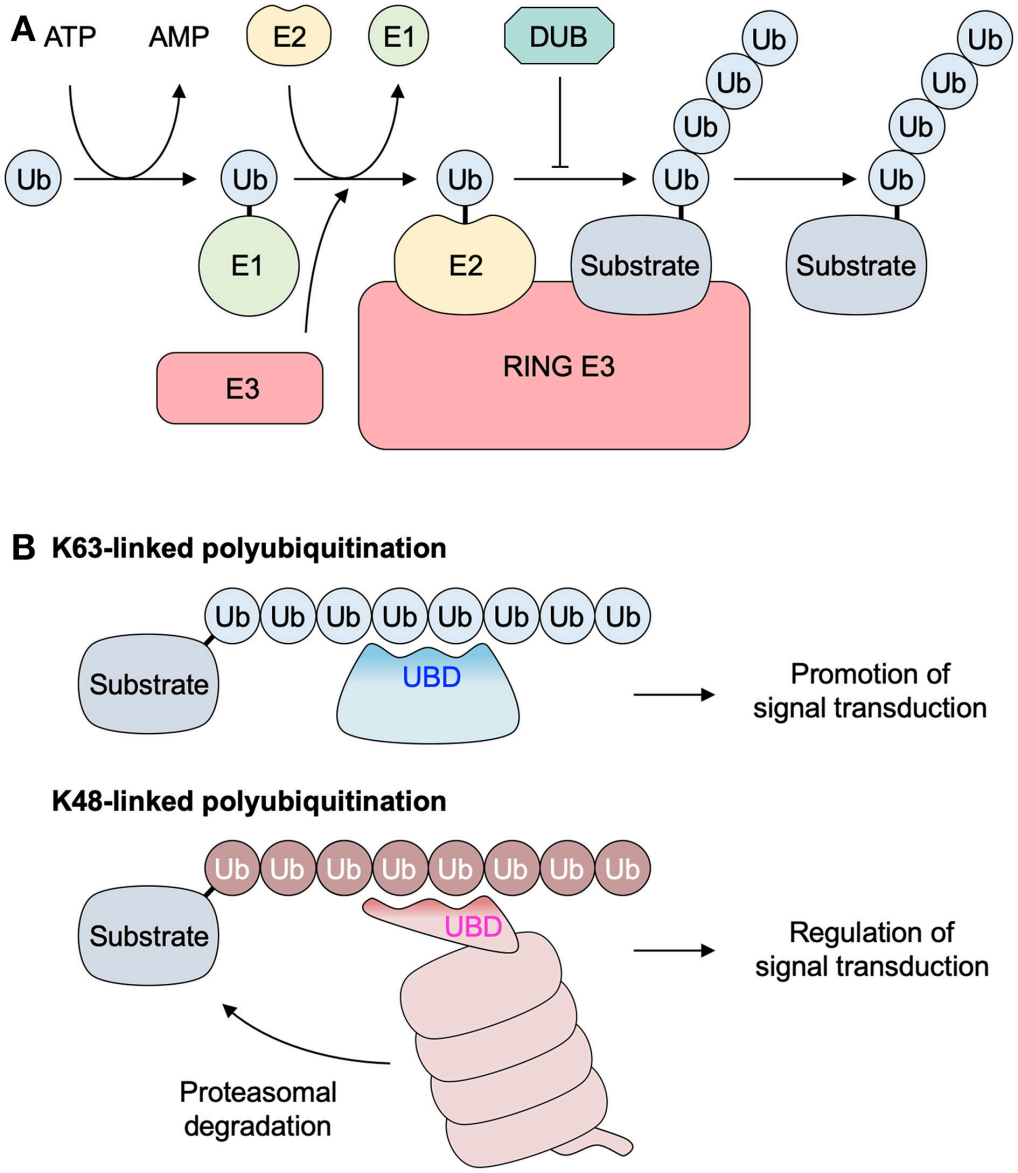
Polyubiquitination system. **(A)** Three enzymes catalyze the polyubiquitination of the substrate protein through a cascade of reactions: a ubiquitin-activating enzyme (E1), a ubiquitin-conjugating enzyme (E2), and a ubiquitin-protein ligase (E3). The E1 enzyme first forms a thiol ester bond with a ubiquitin. The activated ubiquitin is transferred to the E2. A RING E3 enzyme (such as TRAF6) functions as a scaffold for the binding of both the E2 and the target molecule, and facilitates the transfer of ubiquitin from the E2 to the target protein. **(B)** Lys63 (K63)-linked polyubiquitination promotes intracellular signal transduction via the association between a substrate protein with K63-linked ubiquitin chains and a protein with a UBD. K48-linked ubiquitin chains are recognized by proteasome, and subsequent proteasomal degradation of the substrate protein is involved in regulation of intracellular signal transduction in several ways. DUB, deubiquitinase; RING, really interesting new gene; Ub, ubiquitin; UBD, ubiquitin binding domain.

TRAF6 was identified for the first time in 1996 as the new TRAF family member that mediates IL-1 signaling (5) as well as CD40 signaling (6). TRAF6 is a signaling adaptor functioning as an E3 ubiquitin ligase. Ubiquitin signaling is mainly mediated by the ubiquitin conjugation system that conjugates polyubiquitin chains (ubiquitin polymers) to proteins (7). This conjugation is mediated through a cascade of reactions catalyzed by 3 enzymes: a ubiquitin-activating enzyme (E1), a ubiquitin-conjugating enzyme (E2), and a ubiquitin–protein ligase (E3) (Figure 1A). The E3 ligase functions as a scaffold for the binding of both the E2 and target molecule and facilitates the transfer of ubiquitin from the E2 to the target protein. A Ubc13–Uev1a E2 complex generates Lys63 (K63)-linked polyubiquitin chains together with the RING E3 ligase, such as TRAF6. Binding K63-linked polyubiquitin chains to the target molecules plays a crucial role in a variety of immunological functions via regulating intracellular signal transduction (8–10). While various types of polyubiquitin chains are involved in the ubiquitin signaling, the role of K63-linked polyubiquitin chains are well-characterized in nuclear factor κB (NF-κB) pathways (7). K63-linked chains recruit proteins through their selective binding of a ubiquitin-binding domain (UBD) whereas K48-linked chains induce the proteasomal degradation of the substrate proteins regulating signal transduction (Figure 1B).

### Phenotypes of TRAF6 Deficient Mice

TRAF6-deficient (*Traf6*^−/−^) mice appear normal at birth but become progressively runted, and typically die by 3 weeks of age (11–13). Therefore, TRAF6, as well as TRAF2 and TRAF3 (14, 15), is essential for perinatal and postnatal survival. *Traf6*^−/−^ mice exhibit severe osteopetrosis, thymic atrophy, lymph node deficiency, and splenomegaly (11, 12). Spleens from *Traf6*^−/−^ mice are markedly disorganized, with a complete lack of normal T and B cell areas. Small clumps of lymphocytes are scattered throughout splenic sections, but distinct peri-arteriolar or lymphoid collections are absent. Assays *in vitro* demonstrated that TRAF6 is crucial not only in IL-1 and CD40 signaling but also in lipopolysaccharide (LPS) signaling (13). These findings established unexpectedly diverse and critical roles for TRAF6 in perinatal and postnatal survival, bone metabolism, innate immune responses, and cytokine signaling. Further investigation using conditional gene knockout techniques has clarified the immunological phenotypes of TRAF6 deficiency in each immune and epithelial cell subset (described and discussed in chapters 4 and 5, respectively).

## Upstream Molecules

**Figure 2:**
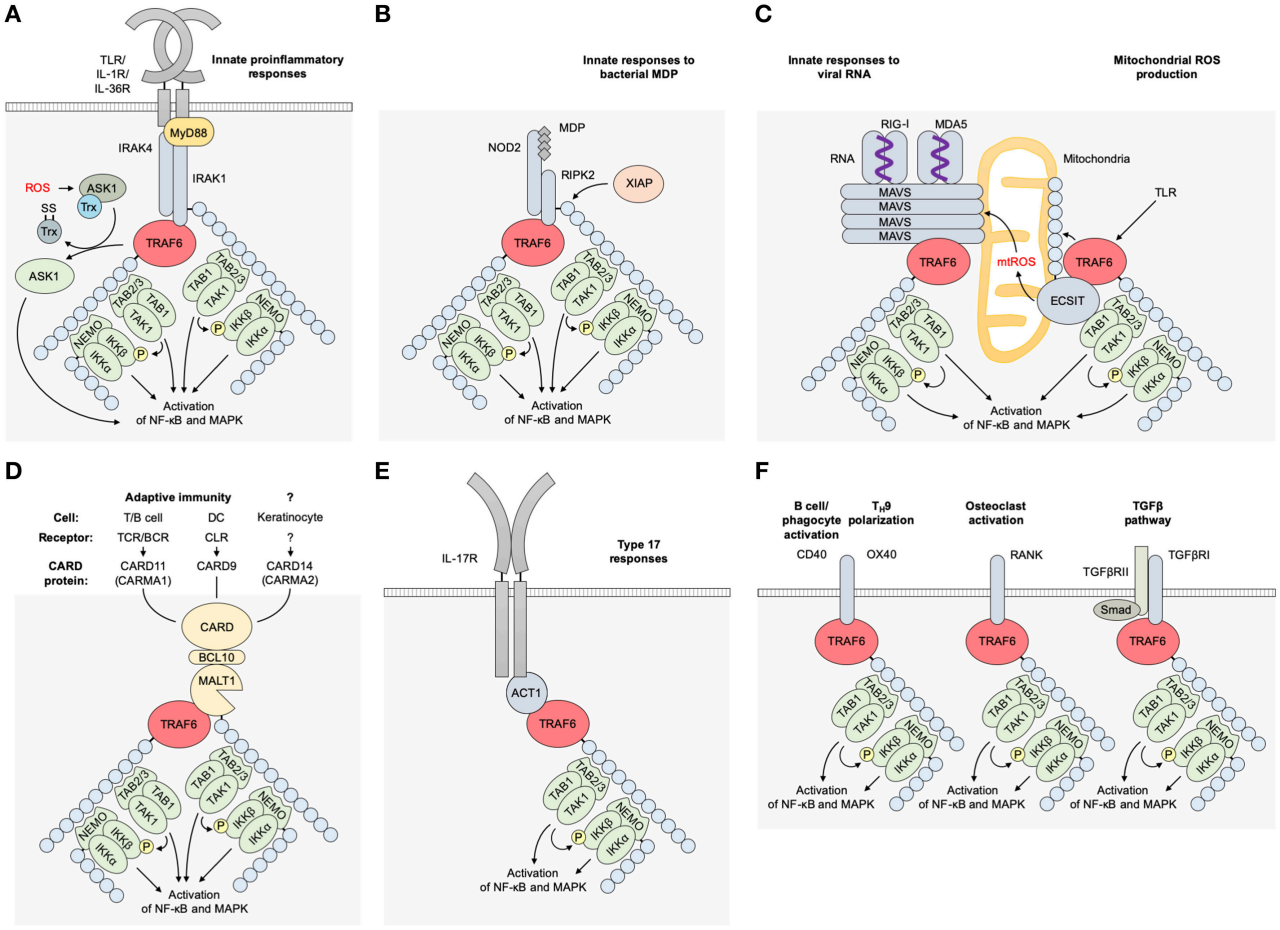
Receptor signaling pathways upstream of TRAF6. **(A)** TLR/IL-1 family pathways. Receptor–ligand bindings cause the association between IRAK4/1 and TRAF6 and subsequent activation of TRAF6 in a MyD88-dependent manner. TRAF6 E3 activity mediates K63-linked ubiquitination of IRAK1, NEMO/IKKγ, and TRAF6 itself, resulting in the activation of NF-κB and MAPKs. **(B)** An NLR pathway. The binding of bacterial MDP or viral RNAs to NOD2 results in the association between RIPK2 and TRAF6, and subsequent activation of TRAF6. K63-linked ubiquitination of RIPK2 is expected to be mediated by another E3 ligase XIAP. **(C)** An RLR pathway. The binding of viral RNAs to RIG-I or MDA5 mediates MAVS polymerization at mitochondria and subsequent binding and activation of TRAF6. mtROS is also involved in the MAVS polymerization. TLR signaling mediates mtROS production via TRAF6 mitochondrial translocation and subsequent binding and polyubiquitination of ECSIT. **(D)** CBM signalosome complex-dependent pathways. The formation of a CBM complex is triggered by activation of CARD proteins: CARD11 (CARMA1) in the TCR/BCR pathway in T/B cells, respectively; CARD9 in the CLR pathway in DCs; and CARD14/CARMA2 in keratinocytes although its upstream receptor remains unidentified. TRAF6 is associated with the CBM complex, and TRAF6 E3 ligase activity mediates K63-linked ubiquitination of MALT1, NEMO/IKKγ, and TRAF6 itself. **(E)** An IL-17 pathway. The ligation of IL-17 cytokines to IL-17R recruits ACT1, which bridges the IL-17R and TRAF6 and promotes the E3 ligase activity of TRAF6. ACT1 also associates with BAFFR in B and T cells and CD40 in B cells and phagocytes, and is expected to regulate these receptor signaling pathways. **(F)** Other TRAF6-dependent pathways. A CD40 pathway in B cells, phagocytes and other cells; an OX40 pathway in T cells; a RANKL pathway in osteoclasts; and a TGFβRI pathway in various cells. ACT1, NF-κB activator 1; ASK1, apoptosis signal-regulating kinase 1; BCL10, B-cell lymphoma/leukemia 10; CARD, caspase recruitment domain-containing protein; DC, dendritic cell; ECSIT, evolutionarily conserved signaling intermediate in Toll pathways; IKK, IκB kinase; IRAK, interleukin-1 receptor-associated kinase, MALT1, mucosa associated lymphoid tissue lymphoma translocation gene 1; MAVS, mitochondrial antiviral signaling protein; MDA5, melanoma differentiation-associated gene 5; MDP, muramyl dipeptide; mtROS, mitochondrial reactive oxygen species; MyD88, myeloid differentiation primary response protein 88; NEMO, NF-κB essential modulator; NF-κB, nuclear factor κB; NLR, NOD-like receptor; NOD, nucleotide-binding oligomerization domain; RANK, receptor activator of NF-κB; nucleotide-binding oligomerization domain; RANKL, RANK ligand; RIG-I, retinoic-acid-inducible gene-I; RLR, RIG-I-like receptor; TAB, TAK1 binding protein; TAK1, transforming growth factor-β-activated kinase 1; TGFβRI, transforming growth factor-β receptor I; TLR, Toll-like receptor; TRAF6, tumor necrosis factor receptor associated factor 6; XIAP, X-linked inhibitor of apoptosis.

TRAF6 is a transducer of a number of receptor signaling pathways. In these pathways, there are TRAF6-binding motifs in the signaling adaptors and receptor molecules, such as IL-1 receptor-associated kinases (IRAKs), mucosa associated lymphoid tissue lymphoma translocation gene 1 (MALT1), mitochondrial antiviral signaling protein (MAVS), NF-κB activator 1 (ACT1), CD40, and receptor activator of NF-κB (RANK) (9, 16) (Figure 2).

### IL-1 and TLR Pathways

The roles of TRAF6 in the MyD88-dependent pathways, such as IL-1 and Toll-like receptor (TLR) pathways, have been extensively investigated (17) (Figure 2A). Upon ligand binding, the IL-1 receptor (IL-1R) and MyD88-dependent TLRs (TLR1, 2, 4, 5, 6, 7, 8, 9, 11, 12, 13) recruit IRAKs via the adaptor MyD88 to trigger the recruitment of TRAF6 and subsequent formation of receptor-associated signaling complexes and ubiquitination of the components. In Toll–IL-1 receptor domain-containing adaptor inducing interferon-β (TRIF)-dependent TLR pathways (such as those of TLR3 and TLR4), TRAF6 is recruited to TRIF and receptor-interacting protein kinase (RIPK)1 kinase, which activates TGF-β-activated kinase 1 (TAK1) in response to TRAF6 activation (18) while TRAF3 has a more important role than TRAF6 in TRIF-dependent signaling (19).

The production of IL-1β requires 2 signals: the priming signal 1 that induces the transcription of IL-1β and nucleotide-binding oligomerization domain (NOD)-like receptor protein 3 (NLRP3), and the activating signal 2 that primes NLRP3 inflammasome and subsequent IL-1β maturation through their processing cascades. In addition to the transduction of the signal 1, it has been reported that TRAF6 is involved in signal 2 (20). TRAF6 promotes NLRP3 oligomerization as well as the interaction between NLRP3 and apoptosis-associated speck-like protein containing a caspase recruitment domain-containing protein (CARD) (ASC) in its ubiquitin E3 ligase activity-dependent manner. Deficiency of TRAF6 specifically inhibits IL-1/TLR priming-initiated caspase-1 cleavage, pyroptosis, and secretion of presynthesized IL-18 (20).

As well as phagocytes and immune cells, epithelial cells express IL-1R family receptors (21) and most TLRs (22, 23). Epithelial cell-specific deletion of *Myd88* has demonstrated intrinsic roles of epithelial IL-1 and TLR pathways in host defense (24–27) and carcinogenesis (28, 29).

### NLR and RLR Pathways

NOD-like receptors (NLRs) recognize bacterial muramyl dipeptide (MDP) and viral RNAs (30) and activate NF-κB via promoting TRAF6 to enhance NF-κB essential modulator (NEMO)/ IκB kinase (IKK) γ polyubiquitination (31) whereas TRAF2/5, but not TRAF6, are essential in NOD1/2-mediated NF-κB activation (32) (Figure 2B). In cytosolic retinoic-acid-inducible gene-I (RIG-I)-like receptor (RLR) pathways, the binding of RIG-I to viral RNAs induces its oligomerization with MAVS that recruits TRAF6 and triggers the activation of the downstream signaling pathways (33) (Figure 2C). It has been demonstrated that double-stranded (ds) RNA induces an antiviral defense status in epidermal keratinocytes through MDA5/RIG-I-mediated signaling (34). In addition, keratinocyte MAVS pathway is activated by a cathelicidin-derived antimicrobial peptide LL37 and dsDNA and involved in interferon (IFN)-β expression in psoriasis and during wound repair (35).

### Mitochondrial ROS Production

TRAF6-mediated mitochondrial reactive oxygen species (mtROS) production is well-demonstrated in macrophages: mtROS production triggered by TLR signaling involves the translocation of TRAF6 to mitochondria, where it engages and ubiquitinates ECSIT (36) (Figure 2C). This process is necessary for the increase in mtROS production. By LPS stimulation, ECSIT forms a complex with TRAF6 and TAK1 leading to the activation of NF-κB (37). Consistently, ECSIT- or TRAF6-deficient macrophages exhibit decreased levels of TLR-induced ROS and defective intracellular bacteria killing (36). It has also been demonstrated that TRAF6 is involved in mtROS production and subsequent apoptosis in human intestinal epithelial cell line Henle-407, and a Salmonella protein SopB binds to TRAF6 and prevent ROS-induced apoptosis of epithelial cells by retarding TRAF6 recruitment to mitochondria (38). In addition, oxidative stress-induced activation of apoptosis signal-regulating kinase 1 (ASK1) and subsequent activation of the MAPK pathway depends on TRAF6 (39). However, the mechanism of TRAF6 mitochondrial translocation or its interaction with a ROS–ASK1–TRAF6 pathway remain enigmatic (9, 40). The RLR signaling is in part potentiated by mtROS induction (40) (Figure 2C).

### CBM Complex

The signalosome-dependent pathways that include T cell receptor (TCR), B cell receptor (BCR), and C-type lectin receptor (CLR) pathways are mediated by the formation of signalosomes — CARD–BCL10–MALT1 (CBM) complexes (41–43). TCR and BCR pathways signal via CARD11/CARD-containing MAGUK protein 1 (CARMA1) while CLR pathways signal via CARD9 (Figure 2D). However, it remains unclear whether the formation of CBM complex is involved in TLR pathways as deficiency in B-cell lymphoma/leukemia 10 (BCL10) or caspase 8, which takes part in the formation of CBM complex, but not MALT1, abolishes the LPS-induced NF-κB activation (44). The CBM complex of CARD14/CARMA2 is expected to bind with TRAF6 and get involved in IL-17 pathways in keratinocytes whereas the upstream receptors of the CARD14 remain unknown (45). The formation of the CBM complexes results in the TRAF6 recruitment, which facilitates the polyubiquitination of the components of CBM complexes and their downstream molecules.

### IL-17 Pathways

The binding of IL-17A and/or IL-17F to the heterodimeric IL-17R leads to the recruitment of ACT1, which allows the incorporation of TRAF6 into the ACT1–TRAF6 signaling complex and then “downstream” activation of NF-κB and mitogen-activated protein kinase (MAPK) pathways (46–48) (Figure 2E). The IL-17R family and ACT1 share sequence homology in their intracellular region with Toll-IL-1 receptor (TIR) domains, and it is involved in their homotypic interaction (46, 49). ACT1 binds to TRAF6 effectively among TRAF family proteins (50). The formation of the IL-17-mediated ACT1–TRAF6 complex is required for IL-17-mediated NF-κB and c-Jun N-terminal kinase (JNK) activation but not for extracellular signal-regulated kinase (ERK) (p44/ERK1/MAPK3 and p42/ERK2/MAPK1) activation (51), or p38-mediated *Cxcl1* mRNA stabilization (52), which indicates the existence of an IL-17-induced and ACT1-mediated but TRAF6-independent pathway. The epithelial IL-17 pathway is expected to organize a unique “loop” in the EIME (discussed in chapter 9).

### Others

Other upstream molecules of TRAF6 have essential roles mainly in non-epithelial cells. CD40 and RANK directly recruit TRAF6 upon the activation of their receptor signaling pathways (9). TRAF6 directly interacts with transforming growth factor (TGF)-β receptor I (TGFβRI) and mediates Smad-independent activation of downstream pathways (53, 54). TRAF6 also functions as an inhibitor of TGF-β-induced Smad2/3 activation in the TGFβR pathway (55). In T_H_9 differentiation, an OX40–TRAF6 binding promotes the TRAF6 E3 ligase activity resulting in non-canonical NF-κB activation (9) (Figure 2F).

## TRAF6 in Immune Cells, Phagocytes and Blood Cells

### Dendritic Cells

TRAF6 regulates the critical processes required for maturation, activation, and development of dendritic cells (DCs) (13). In response to LPS or CD40 stimulation, TRAF6-deficient DCs fail to upregulate surface expression of major histocompatibility complex (MHC) class II and B7.2, or to produce inflammatory cytokines. In addition, LPS-treated TRAF6-deficient DCs do not exhibit an enhanced capacity to stimulate naive T cells while splenic DC development is severely impaired as the CD4+CD8α- splenic DC subset is nearly absent in TRAF6-deficient mice (13).

TRAF6 in DCs has been shown to be critical for gut microbiota-dependent immune tolerance (56). DC-specific deletion of TRAF6 in *CD11c-Cre Traf6* ^*flox*/*flox*^ mice leads to diminishing gut commensal microbiota-dependent DC expression of IL-2 and results in reduced numbers of regulatory T (Treg) cells associated with spontaneous development of T_H_2 cells, eosinophilic enteritis, and fibrosis in the small intestine (56).

### T Cells

Generation of *CD4-Cre Traf6* ^*flox*/*flox*^ mice made specific deletion of TRAF6 in T cells possible (both in CD4+ T cells and CD8+ T cells at the CD4+ CD8+ double positive stage during T cell development) (57). TRAF6-deficient T cells exhibit hyperactivation of a phosphatidylinositol 3 kinase (PI3K)–Akt pathway compared with wild-type T cells and become resistant to suppression by CD4+CD25+ Treg cells (57). In addition, TRAF6-deficient CD8+ T cells exhibit altered metabolism of fatty acids, such as metformin. As a result, T cell-specific deletion of TRAF6 generates defective long-lived memory CD8+ T cells, which are rescued by metformin treatment (58).

### B Cells

TRAF6 is originally identified as the TRAF family protein that directly associates with the cytoplasmic region of CD40 and its intracellular signaling and thus that plays crucial roles in B-cell function (6). The CD40–TRAF6 binding is important for IL-6 production, upregulation of CD80/B7.1, IL-6-dependent production of immunoglobulin by B cells (59), and subsequent affinity maturation and generation of long-lived plasma cells (60). Also, TRAF6 mediates T cell-independent (CD40-independent) immunoglobulin responses. The transmembrane activator TACI triggers immunoglobulin class switching by activating B cells through a TLR-like MyD88–TRAF6 pathway (61). Consistent with these results, B cell-specific deletion of TRAF6 in *CD19-Cre Traf6* ^*flox*/*flox*^ mice results in a reduced number of mature B cells in the bone marrow and the spleen, impaired T cell-dependent and independent immunoglobulin responses, and defective generation of B-1 B cells (62).

### Macrophages

CD40–TRAF6 signaling in macrophages mediates downstream activation of IKK–NF-κB and ERK MAPK pathways, which are involved in many phagocytic functions, such as IL-12 induction, autophagic vacuole–lysosome fusion in synergy with TNF signaling, and atherogenesis (9). Besides, TLR/RLR–TRAF6 signaling in macrophages induces the production of mtROS (described in section 3.3).

### Osteoclasts

RANK ligand (RANKL)–TRAF6 signaling is critical for osteoclast development and maintenance via the activation of NF-κB, MAPK, and Akt pathways, in addition to the expression of nuclear factor of activated T cells cytoplasmic 1 (NFATc1), which is an osteoclast master regulatory transcription factor (9).

### Hematopoietic Stem Cells

TRAF6-dependent basal NF-κB activation is required for hematopoietic stem cell homeostasis in the absence of inflammation (63).

## TRAF6 Signaling Pathways in Barrier Tissues

### TRAF6 in the Skin

The skin is the outer protective wrapping of the body and continuously defends against external dangers and pathogens (1). The epidermis is the outermost layer of the skin that acts as a physical barrier and regulator of the protective responses (1). Epidermal keratinocytes express TRAF6, which participates in many intracellular signaling pathways, such as TLR pathways, IL-1 pathways, and IL-17 pathways; all of which are involved in the host defense system and inflammatory processes.

Results of animal experiments suggest that epidermal TRAF6 is required for the initiation and propagation of IL-17-mediated psoriatic inflammation (3). The development of psoriatic dermatitis induced by topical imiquimod is abolished in *K5-Cre Traf6* ^*flox*/*flox*^ mice lacking TRAF6 in keratinocytes (3). TRAF6 depletion in keratinocytes impairs subsequent activation of skin resident DCs and their production of IL-23, and hinders IL-17A production of Vγ4+ γδ T cells in the skin. Moreover, TRAF6-null keratinocytes were resistant to the stimulation with either imiquimod or IL-17 *in vitro*, with subsequent absence of their psoriasis mediators for DC recruitment and activation. These results suggest that keratinocyte TRAF6 machinery is required for both the primary response to imiquimod and the secondary responses to IL-17 cytokines produced by T cells in this animal model. This is consistent with the idea that keratinocytes have critical roles in both the primary response to external dangers and the secondary propagation of inflammatory loop mediated by IL-17 cytokines in psoriasis (1).

### TRAF6 in the Gut

Mice lacking TRAF6 in intestinal epithelial cells (IECs) (*Villin-Cre Traf6* ^*flox*/*flox*^) show an exacerbated phenotype in DSS colitis: a model for intestinal bowel diseases (4). On the other hand, depletion of TLR signaling in IECs by ablation of MyD88 and TRIF in *Villin-Cre Myd88* ^*flox*/*flox*^ *Ticam1* ^*flox*/*flox*^ mice does not affect the severity of DSS colitis (4). In addition, germfree mice are known to be more susceptible to DSS colitis (64). These findings suggest that microbiota–host interactions may control the intestinal homeostasis, and TLR-independent intestinal epithelial TRAF6 signaling could have a beneficial role in this animal model.

### TRAF6 in Epithelial Primary and Secondary Responses

During host protection and inflammation, epithelial cell responses are divided into 2 phases: (i) primary responses to external triggers and (ii) secondary responses to internal immune mediators (1). Studies using *K5-Cre Traf6* ^*flox*/*flox*^ mice suggest that TRAF6 governs both the primary and secondary responses of keratinocytes, and the both are required for the initiation and propagation of psoriatic inflammation (3). Both response to imiquimod and IL-17A are defective in TRAF6-null keratinocytes, and compensation of primary responses by subcutaneous injection with IL-23 is not sufficient for the full development of psoriatic inflammation. TLR/IL-1–TRAR6 pathways are expected to trigger the primary responses while IL-17–TRAF6 pathways mediate the secondary responses. In epithelial cells, TRAF6 thus plays a unique role as “a hub” among TLR/IL-1 pathways and IL-17 pathways in the type 17 response by effective protein–protein interaction and synergistic activation of these pathways whereas the precise molecular mechanism remains to be elucidated. Furthermore, one may be tempted to speculate common and fundamental roles for TRAF6 signaling in epithelial cells as discussed in the next section.

### Epithelial TRAF6 in Protective Responses in the EIME

**Figure 3:**
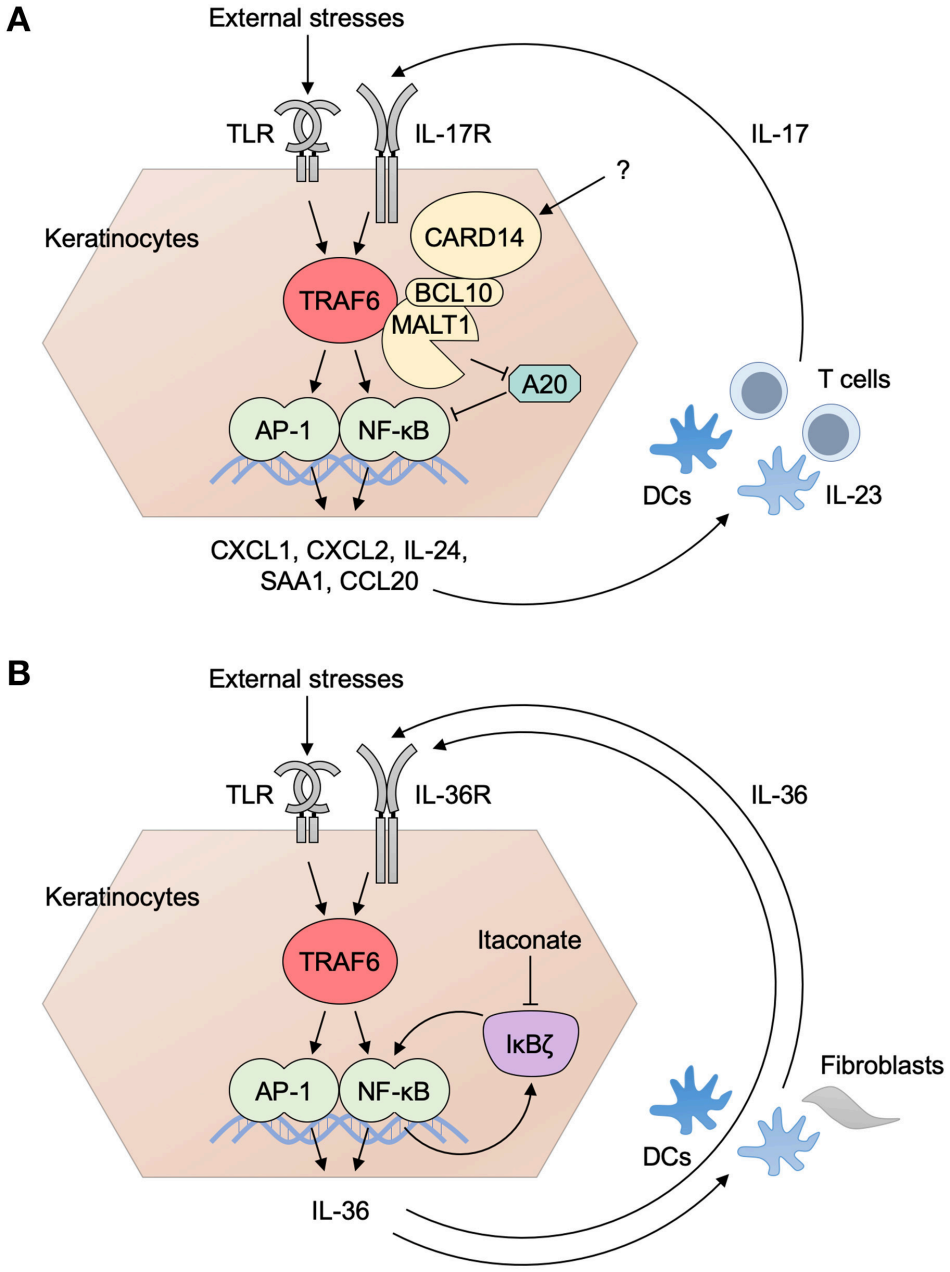
Inflammatory loops in the EIME of psoriasis. **(A)** The inflammatory loop of IL-17 in psoriasis. TRAF6-dependent activation of danger signals (signal 1: such as TLR pathways) induces the production of psoriasis mediator from keratinocytes. Subsequent activation of the IL-23/IL-17 axis in DC–T cell interaction gives rise to the production of IL-17 (signal 2) that drives further activation of keratinocytes. CARD14 associates with ACT1 and TRAF6 and is involved in IL-17 signaling. Activated MALT1 degrades A20 and suppresses its regulatory roles for ubiquitin signaling. **(B)** The inflammatory loop of IL-36 and IκBζ in psoriasis. IL-36R is an IL-1R family receptor and its signaling pathway is expected to be TRAF6-dependent. The activation of an IL-36 pathway by the ligation of IL-36 to its receptor triggers the expression of IκBζ. It promotes the transcriptional expression of itself, as well as that of a series of psoriasis mediators inducing the production of IL-17 from immune cells. IL-36 from keratinocytes binds to IL-36R in keratinocytes and other cells, such as DCs and fibroblasts, and drives the loop of IL-36 pathway. Transcriptional regulation by IκBζ is in part mediated by histone methylation. Itaconate inhibits the protein induction of IκBζ and ameliorates psoriatic inflammation. AP-1, activator protein 1; BCL10, B-cell lymphoma/leukemia 10; CARD, caspase recruitment domain-containing protein; CXCL, CXC chemokine ligand; CCL, CC chemokine ligand; DC, dendritic cell; MALT1, mucosa associated lymphoid tissue lymphoma translocation gene 1; NF-κB, nuclear factor κB; SAA, serum amyloid A; TLR, Toll-like receptor; TRAF6, tumor necrosis factor receptor associated factor 6.

The results of epithelial TRAF6 depletion in the skin and in the gut are seemingly opposing because epidermal TRAF6 depletion results in the abolishment of imiquimod-induced inflammation (3) whereas IEC-specific TRAF6 depletion results in exacerbation of DSS-induced inflammation (4). However, IL-17 cytokines have major protective roles in mucocutaneous fungal infections (65–67) although they are related to the pathogenesis of psoriasis and its animal models. On the other hand, intestinal barrier integrity is maintained by IL-17 cytokines (68) and the IL-17 cytokines are related to the reduction of DSS colitis (69). Thus, it is a plausible idea that epithelial TRAF6 is uniquely involved in local, IL-17-mediated, protective responses in the skin and the gut despite its pathogenetic role in IL-17-mediated psoriatic inflammation (Figure 3A).

Collectively, epithelial TRAF6 is expected to have a pivotal role in the initiation and propagation of type 17 immune and protective responses, which are required at the outermost part of the body and distinctive in epithelial tissues. Especially, its involvement in the secondary responses to internal immune mediators characterizes the definitive role of epithelial TRAF6 in the EIME. In turn, TRAF6 is not just involved in homeostatic barrier protection and host defense, but also can be involved in the chronic inflammation via driving a “loop” of inflammation, as discussed at the latter part of this review.

## Downstream Effectors of TRAF6

**Figure 4:**
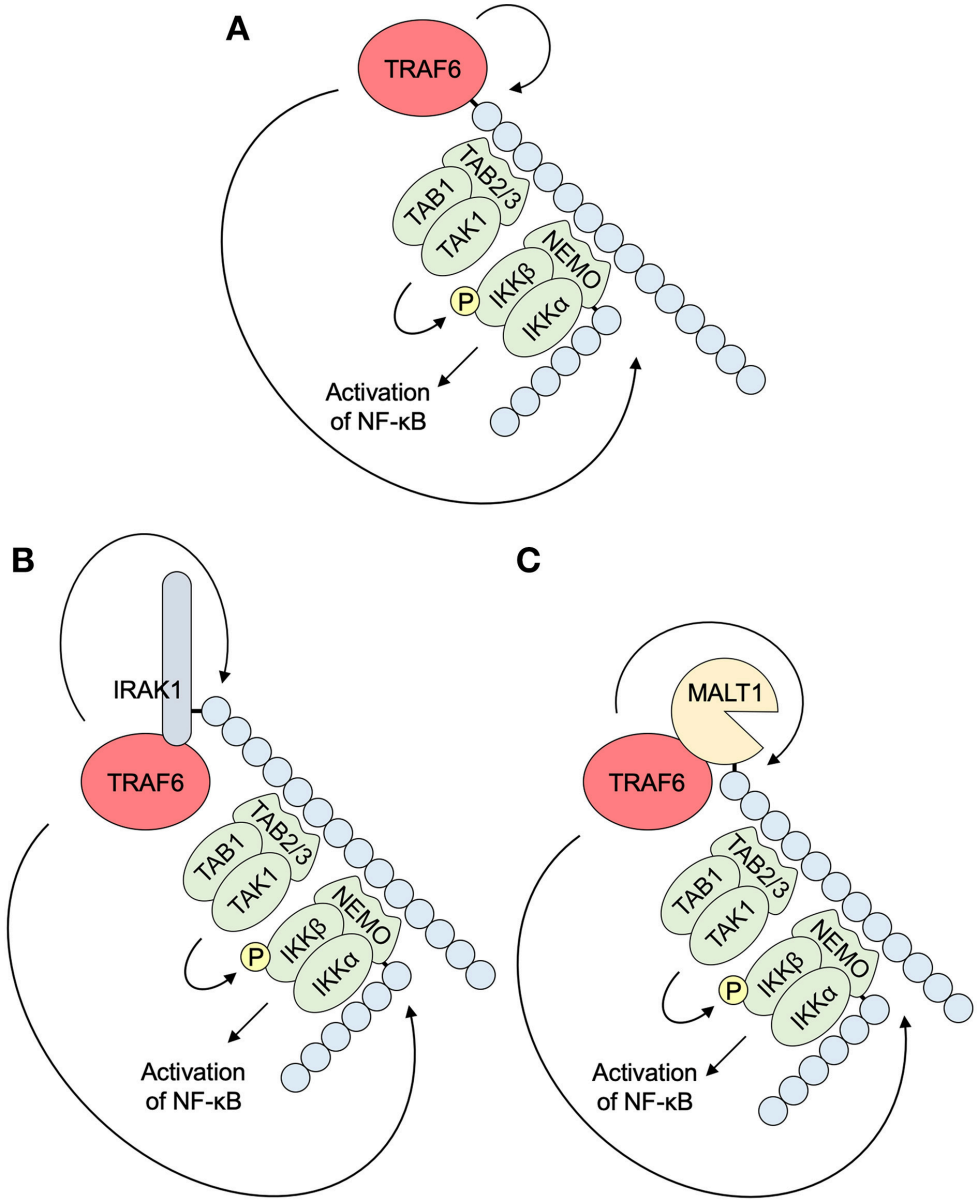
NF-κB pathways downstream of TRAF6. **(A)** K63-linked self-ubiquitination by E3 ligase activity of TRAF6 accumulates TAB2/3 with a UBD and triggers subsequent association and activation of TAB1 and TAK1. NEMO/IKKγ also binds to K63-linked ubiquitin chains via a UBD, and associates with IKKα and IKKβ. NEMO ubiquitination by TRAF6 promotes the formation of an IKK complex. TAK1 activation is followed by the activation of MAPK pathways. IKKβ phosphorylation by TAK1 results in the activation of NF-κB pathways. **(B)** TRAF6 E3 ligase activity polyubiquitinates IRAK1, an upstream molecule of TRAF6 in IL-1/TLR pathways (see **A**). **(C)** TRAF6 E3 ligase activity polyubiquitinates MALT1, a component of a signalosome in TCR/BCR pathways, CLR pathways, and others (see Figure 2D). IKK, IκB kinase; IRAK, interleukin-1 receptor-associated kinase; MALT1, mucosa associated lymphoid tissue lymphoma translocation gene 1; NEMO, NF-κB essential modulator; NF-κB, nuclear factor κB; TAB, TAK1 binding protein; TAK1, transforming growth factor-β-activated kinase 1; TRAF6, tumor necrosis factor receptor associated factor 6.

**Figure 5:**
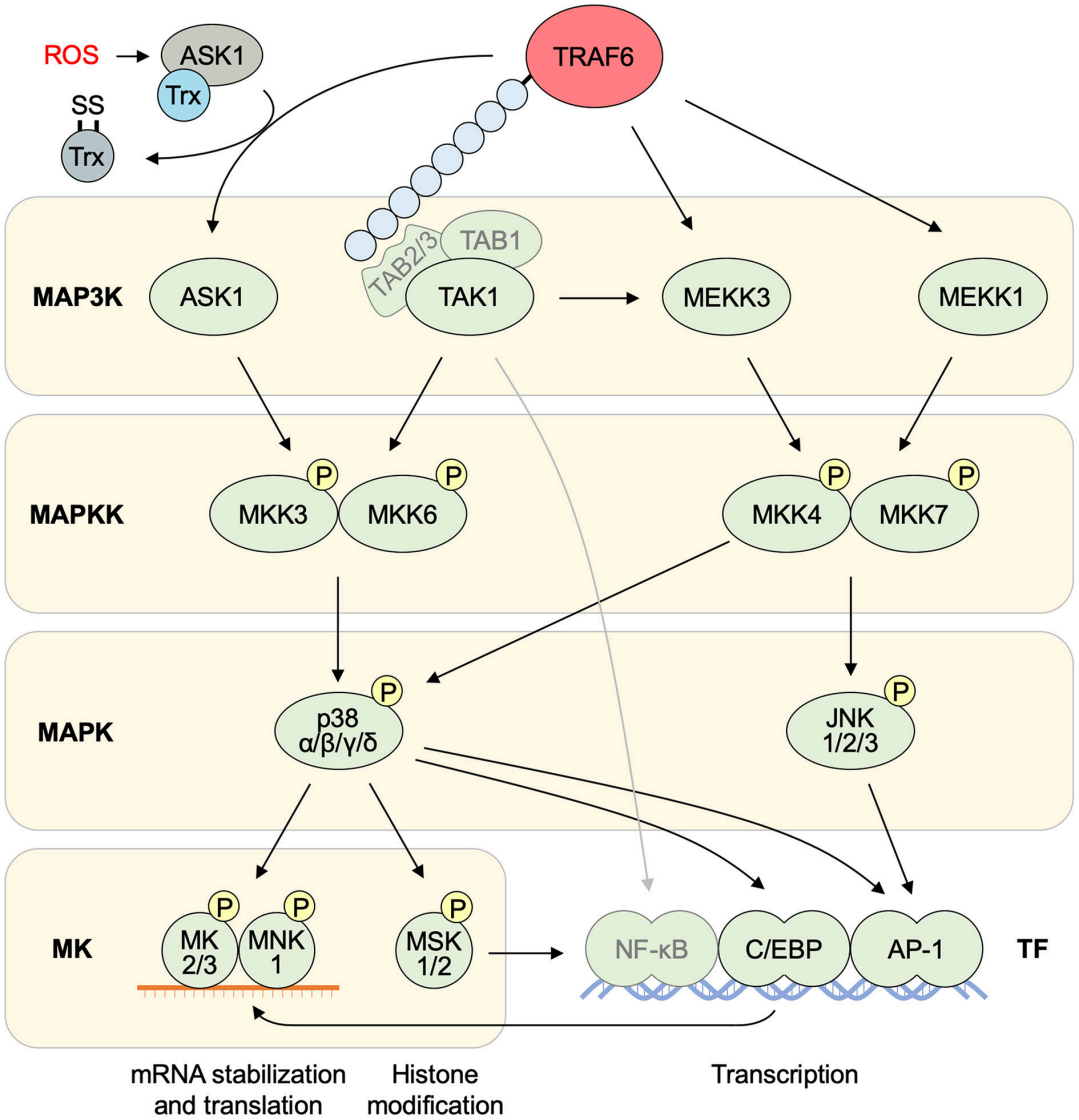
MAPK pathways downstream of TRAF6. MAPK pathways are regulated by the phosphorylation cascade of MAP3Ks, MAPKKs, and MAPKs, resulting in the activation of MKs and a transcription factor AP-1. TRAF6 mediates the activation of MAP3Ks that are mainly involved in p38 and JNK MAPK activation. TRAF6 activates ASK1 MAP3K under oxidative stresses by removing thioredoxin. TRAF6-mediated K63-linked ubiquitin chains recruit TAB2/3 and subsequent activation of TAK1, which is also involved in the activation of NF-κB pathways. ASK1 and TAK1 are involved in the activation of p38 MAPK. p38 regulates gene expression at both transcriptional and post-transcriptional levels. C/EBP is a major transcription factor downstream of p38 MAPK. The E3 ligase activity of TRAF6 is required for the activation of other MAP3Ks MEKK3 and MEKK1, which are mainly involved in the activation of JNK MAPK, resulting in AP-1-mediated gene transcription. AP-1, activator protein 1; ASK1, apoptosis signal-regulating kinase 1; ERK, extracellular signal-regulated kinase; JNK, c-Jun N-terminal kinase; MAPK, mitogen-activated protein kinase; MAPKK, MAPK kinase; MAP3K, MAPK kinase kinase; MEKK; MAPK/ERK kinase kinase; MK, MAPK-activated protein kinase; MKK, MAPK kinase; MNK, MAPK-interacting kinase; MSK, mitogen- and stress-activated kinase; NF-κB; ROS, reactive oxygen species; NF-κB, nuclear factor κB; TAB, TAK1 binding protein; TAK1, TGF-β-activated kinase 1; TRAF6, tumor necrosis factor receptor associated factor 6; Trx, thioredoxin.

NF-κB pathways (Figure 4) and MAPK pathways (Figure 5) are the major downstream effectors of TRAF6 in epithelial cells (8–10).

### NF-κB

NF-κB is the ubiquitous and inducible transcription factor that induces host and cell-protective responses (70). Upon activation, TRAF6 catalyzes the generation of K63-linked polyubiquitin chains on itself (Figure 4A), or other target proteins, such as IRAK1 (Figure 4B), MALT1 (Figure 4C), and NEMO/IKKγ (Figures 4A–C). These chains recruit TAK1 binding protein (TAB) 2/3 that contains a UBD. TAB2/3 in turn recruits ubiquitin-dependent kinase TAK1. Its downstream kinase IKKγ also has an UBD and is recruited to the K63-linked chains and forms an IKK complex with IKKα and IKKβ. These events assemble a signaling complex that facilitates TAK1 and IKK activation. TAK1 phosphorylates and activates IKKβ, which activate a transcription factor, NF-κB (7, 10, 71). TAK1 is also involved in the activation of MAPKs and a transcription factor, activating protein-1 (AP-1), as described in the next section. TRAF6 is also involved in the activation of the NF-κB pathway via the attachment of K63-linked chains to BCL10 and MALT1, which recruit the IKK complex, in CBM signalosome complex-dependent pathways (71, 72) (Figure 4C).

For non-canonical NF-κB activation, TRAF6 is required for the activation of an NF-κB inducing kinase (NIK)-dependent IKKα-RelB-p52 pathway (73). NF-κB pathways can affect the activation of the MAPK pathways, as described below.

### MAPK

MAPK is a kinase family, which includes p38, JNK, and ERK (74, 75). These kinases have distinct roles in cell stress responses and cell proliferation. The MAPK activation is controlled by a three-layered kinase cascade: a MAP kinase kinase kinase (MAP3K), a MAP kinase kinase (MKK), and a MAPK (Figure 5).

TRAF6 is involved in the activation of p38 and JNK through multiple MAP3Ks. ASK1 is a MAP3K of the p38 and JNK MAPK pathways (76, 77). An ASK1–MAPK pathway is preferentially activated in response to various types of cellular stresses. ASK1 forms a complex, which is constitutively inactive by the association with thioredoxin, yet TRAF2 and TRAF6 interact with and activate ASK1. H_2_O_2_-induced ASK1 activation and cell death are strongly reduced in the cells derived from *Traf2*^−/−^ and *Traf6*^−/−^ mice (39). Moreover, TRAF6 is involved in the activation of other MAP3Ks, such as MAPK–ERK kinase kinase (MEKK)1/3 and TAK1. In response to IL-1 and LPS, evolutionarily conserved signaling intermediate in Toll pathways (ECSIT) interacts with TRAF6 and mediates the processing of MEKK1 and subsequent activation of NF-κB and JNK (78). TRAF6 also forms a complex with MEKK3, which activates NF-κB, JNK, and p38 but not ERK (79). TAK1 does not only contribute to NF-κB activation via IKKβ phosphorylation but also to AP-1 activation via an MKK7-JNK pathway (80) and an MKK6-p38 pathway (81). The K63-linked polyubiquitination of TAK1, likely catalyzed by TRAF6, leads to the formation of TRAF6–TAK1–MEKK3 complex resulting in effective activation of TAK1 and MEKK3 whereas MEKK3 can also be activated in a TAK1-independent manner (82).

p38 and JNK control gene transcription via activation of a transcription factor AP-1 while p38 MAPKs control post-transcriptional and epigenetic regulation of gene expression via activation of a set of MAPK-activated protein kinases (MKs): MK2, MK3, and MAPK-interacting kinase (MNK) 1 regulate mRNA stability and translation; mitogen- and stress-activated kinase (MSK) 1 and MSK2 modulate histone modification (74, 75).

### PI3K–Akt Pathway

Several lines of evidence has suggested the links between TRAF6 and phosphoinositide 3-kinase (PI3K)–Akt pathway in various ways. RANKL and CD40 signaling pathway does not only activate NF-κB and MAPK, but also PI3K–Akt pathway via TRAF6 (83, 84): RANK and CD40 recruit TRAF6, Src family kinases, Cbl family-scaffolding proteins, and PI3K in a ligand-dependent manner, resulting Akt activation. TGFβ also activates PI3K-Akt signaling via TRAF6 in prostate cancer cells (85). In addition, LPS-induced activation of Akt depends on TRAF6 in platelets (86). In human airway epithelial cells, it was also demonstrated that two independent signaling pathways are involved in IL-17 signaling: one involves Akt1–TRAF6–TAK1–NF-κB activation, and the other is related to the Janus kinase (JAK)-associated PI3K signaling pathway (87). Studies using kidney epithelial collecting duct cells suggested that TRAF6 mediates K63-linked polyubiquitination and subsequent activation of Akt, which is required for cell adhesion via α3β1 and α6 integrins (88). Of note, however, it remains obscure whether TRAF6-dependent Akt activation has an essential role for epithelial cells in the EIME.

### C/EBP

CCAAT/enhancer binding proteins (C/EBPs) are a family of transcription factors, and have pivotal roles for cellular proliferation and differentiation, metabolism, and inflammation (89). In the cooperative IL-6 gene transcription by IL-17 and TNF in a bone or fibroblast cell line, both the NF-κB and C/EBP sites in the IL-6 promoter are found to be important, and C/EBPδ, and C/EBPβ appeared to be important for this cooperative transcription (90). In human hepatoma cells, IL-17 induces C/EBPβ activation via TRAF6 and TRAF6-dependent p38 MAPK (Figure 5) and ERK1/2 activation (91). C/EBPβ is expressed by terminally differentiated keratinocytes in psoriasis lesional skin and in 3D-cultured human keratinocytes treated with IL-17 (92). On the other hand, studies using stroma cell line ST2 have shown that C/EBPβ phosphorylation by ERK and glycogen synthase kinase 3β (GSK3β) exerts an inhibitory effect on IL-17-induced gene expression (93). In addition, *Cebpb*^−/−^ mice are resistant to IL-17-mediated experimental autoimmune encephalomyelitis (EAE) (94) and susceptible to systemic candidiasis (95) but resistant to oropharyngeal candidiasis (OPC) (95). Specifically, C/EBPβ contributes to immunity to mucosal candidiasis during cortisone immunosuppression in a manner linked to β-defensin 3 expression (95). These findings suggest that the TRAF6–C/EBP pathway is not essential for the expression of some IL-17-response genes, such as *Defb3*, but for others, in the EIME.

In addition to C/EBP, many molecules are involved in the regulation of the TRAF6 pathway in epithelial cells. Particularly, the regulation of the IL-17–TRAF6 pathway has been extensively investigated. In the next chapter, we summarize the regulatory mechanism of epithelial signaling pathway downstream of TRAF6. We focus on the IL-17–TRAF6 pathway, which has a characteristic role in epithelial tissues.

## Regulatory Mechanisms of the IL-17–TRAF6 Pathway

### ACT1

Deficiency of ACT1 in fibroblast results in a selective defect in IL-17-induced activation of an NF-κB pathway and abrogates IL-17-induced cytokine and chemokine expression (49). The N-terminal domain of ACT1 is essential for the interaction with TRAF6 and for IL-17-mediated NF-κB activation in mouse embryonic fibroblasts (MEFs) (96). ACT1-deficient mice develop much less inflammatory disease in both EAE and DSS-induced colitis due to the impaired IL-17-induced expression of inflammation-related genes in ACT1-deficient astroglial cells or gut epithelial cells (46). In humans, a biallelic missense mutation (T536I) in ACT1 abolishes the homotypic interaction between IL-17R and ACT1, resulting in impaired IL-17 responses with chronic mucocutaneous candidiasis (97).

The precise structure of the ACT1–TRAF6 complex remains obscure. The crystal structure of TRAF6 complexed with TRAF6-binding peptides from CD40 and RANK has proposed the TRAF6-binding motif (98). It is shared in CD40, RANK, and IRAK1, yet there are marked structural differences between receptor recognition by TRAF6 and that by other TRAFs (98). In ACT1, three TRAF6-binding motifs have been suggested: at amino acid residues (in human ACT1) 15–20 (96) and 37–42 (50) in the N-terminal region and 327–334 in the Ser–Gly–Asn–His (SGNH) hydrolase region (99).

The contribution of epithelial ACT1 in the development of psoriasis is still a matter of debate. On the one hand, D19 in the N-terminal region is critical for IL-17 signaling and interaction with TRAF proteins, IKKε [also known as IKKi; inducible IKK, discussed in section IKKε (IKKi)], and a chaperone heat shock protein (Hsp)90 (section Hsp90) (100). On the other hand, a D19A mutation is concluded to be a loss-of-function variant associated with psoriasis susceptibility (101–103). Also, ACT1-deficiencient mice spontaneously develop IL-22-dependent dermatitis (100). However, the hyperactive type 17 response related to the D19A mutation seems to be epithelial cell-independent, because mice with a T cell-specific deficiency in ACT1 (*Lck-Cre Traf3ip2* ^*flox*/−^) also developed a hyperactive type 17 response, suggesting a T cell-intrinsic phenotype.

### Other TRAF Proteins

**Figure 6:**
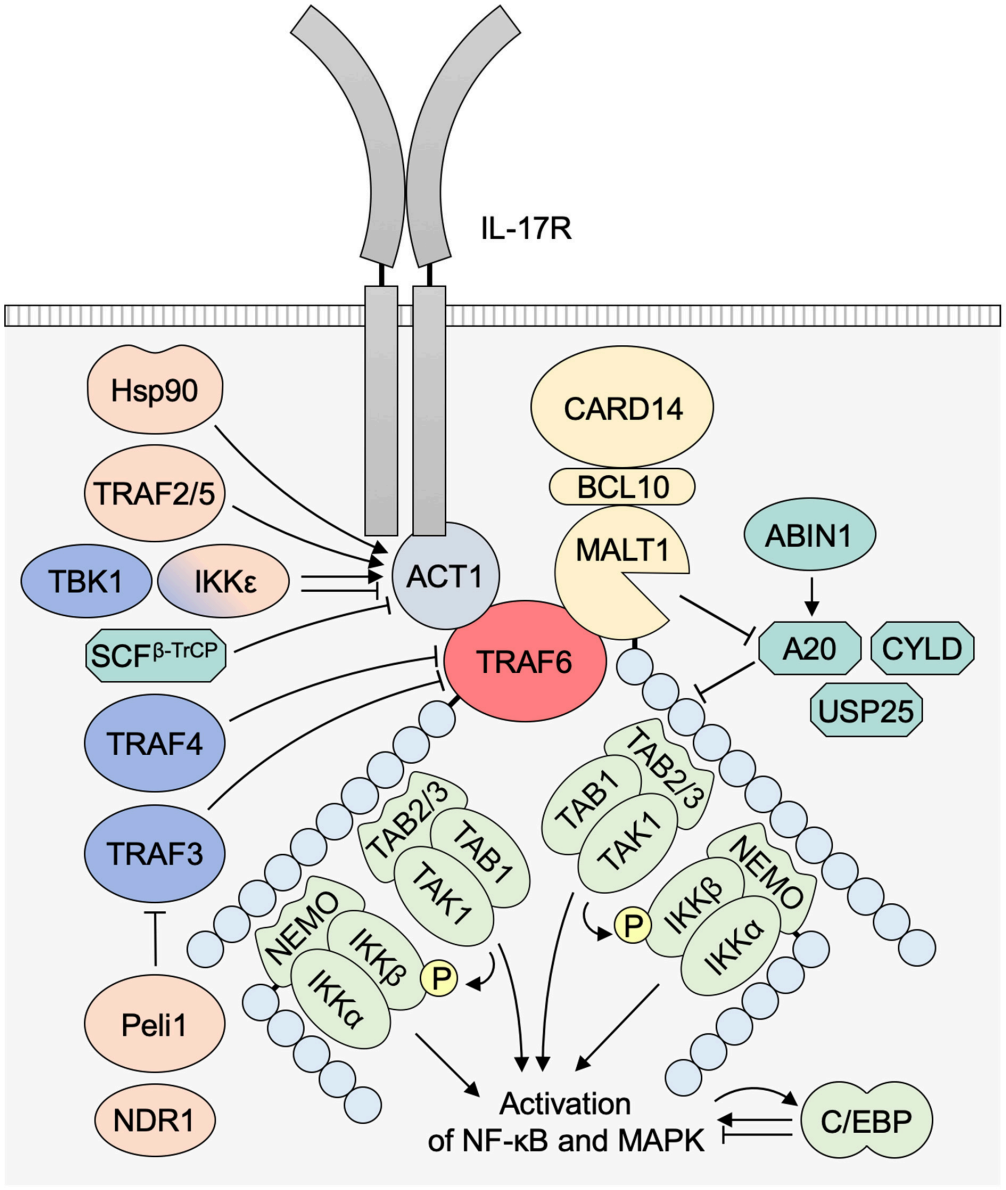
The regulatory mechanisms of the IL-17–TRAF6 pathway. TRAF2 and TRAF5 interact with ACT1 and activate downstream MAPK signaling. The TRAF2/5 binding with ACT1 is dependent on ACT1 S311 phosphorylation by IKKε. IKKε and TBK1 can also be involved in the suppression of the association of ACT1–TRAF6 binding. Hsp90 is a chaperone protein of ACT1. The binding between Hsp90 and ACT1 is required for the IL-17 signaling and is dependent on ACT1 D19. SCF^β−*TrCP*^ E3 ubiquitin ligase complexes are involved in the K48-linked polyubiquitination and degradation of ACT1. TRAF3 is expected to inhibit IL-17 signaling by competing with ACT1 to interact with IL-17R. TRAF4 is also suggested to be a negative regulator for IL-17 signaling, probably due to the competition of TRAF4 with TRAF6 for the interaction with ACT1. Peli1 opposes to TRAF3, by promoting TRAF6-induced K63-linked ubiquitination of c-IAP, which then ubiquitinates TRAF3 with K48 linkage, resulting in TRAF3 proteasomal degradation. NDR1 interferes TRAF3–TRAF6 interaction. A20 is a negative regulator for ubiquitin signaling via dual activity: (1) deubiquitinase activity to K63-linked chains and (2) K48-linked ubiquitinase activity mediating proteasomal degradation of substrate signaling molecules. ABIN1 promotes A20 activity. CYLD and USP25 are other deubiquitinases. C/EBP has both stimulatory and regulatory roles in the transcription of IL-17-response genes. ABIN1, A20 binding and inhibitor of NF-κB-1; ACT1, NF-κB activator 1; BCL10, B-cell lymphoma/leukemia 10; CARD, caspase recruitment domain-containing protein; C/EBP, CCAAT/enhancer binding protein; Hsp, heat shock protein; IKK, IκB kinase; MALT1, mucosa associated lymphoid tissue lymphoma translocation gene 1; NDR1, Nuclear Dbf2-related kinase 1; NEMO, NF-κB essential modulator; NF-κB, nuclear factor κB; SCF^β−*TrCP*^, Skp1-cullin-1-F-box protein β-transducin repeat-containing protein; TAB, TAK1 binding protein; TAK1, transforming growth factor-β-activated kinase 1; TBK1, TANK-binding kinase 1; TRAF, tumor necrosis factor receptor associated factor; USP25, ubiquitin-specific protease 25.

TRAF3 is supposed to have a negative role in IL-17 signaling (104) (Figure 6). Treatment with TRAF3 siRNAs enhances IL-17 signaling in HeLa cells, and exacerbates EAE driven by IL-17 in mice. The enhanced IL-17 signaling in *Traf3*^−/−^ MEFs are reversed by transfection with TRAF3. TRAF3 is assumed to inhibit IL-17 signaling by competing with ACT1 to interact with IL-17R. Nuclear Dbf2-related kinase 1 (NDR1) interacts with TRAF3 and prevents its binding to IL-17R, and consequently, NDR1 functions as a positive regulator of IL-17 signaling (105). The expression of NDR1 in the colon mucosal epithelial cells of ulcerative colitis patients is increased, suggesting the positive regulation of IL-17 signaling and production of inflammatory mediators (105).

TRAF4 is also suggested to be a negative regulator of IL-17 signaling (Figure 6) supported by the evidence that TRAF4 deficiency increases IL-17 signaling in mouse primary kidney cells and exacerbates EAE in mice (106). Therefore, we could conclude a restricting role for TRAF4 in IL-17 signaling, probably due to the competition of TRAF4 with TRAF6 for the interaction with ACT1. Besides, an IL-17-dependent TRAF4-ERK5 axis is suggested to drive a positive feedback loop of p63-mediated TRAF4 expression in keratinocyte proliferation (107).

TRAF2 and TRAF5 interact with ACT1 and activate downstream MAPK signaling (99). The TRAF2/5–ACT1 interaction are dependent on the ACT1 phosphorylation at S311, adjacent to a putative TRAF-binding motif.

### IKKε (IKKi)

IKKε (IKKi) forms a complex with ACT1 and mediates IL-17-induced phosphorylation of ACT1 at S311, which is required for the IL-17-mediated ACT1–TRAF2/5 interaction but not for ACT1–TRAF6 interaction (99) (Figure 6). The IL-17-mediated ACT1 S311 phosphorylation by IKKε and subsequent formation of ACT1–TRAF2/5 interaction is involved in IL-17 signaling. IKKε also participates in a TLR3/4–TRIF–TANK-binding kinase 1 (TBK1) pathway (108). Despite the requirement of IKKε for the ACT1 S311 phosphorylation in IL-17 signaling, IKKε and TBK1 phosphorylate ACT1 in three other Serine sites to suppress the association of ACT1 with TRAF6 and downstream NF-κB activation (109). IKKε-deficiency in airway epithelial cells reduces IL-17-induced JNK and p38 activation, and expression of IL-17-response genes (including *Cxcl1, Cxcl2, and Il6*), suggesting that IKKε is a modulator of IL-17 signaling through its effect on ACT1 phosphorylation and ACT1–TRAF interaction in epithelial cells. However, a precise mechanism for balancing between the ACT1–TRAF6 and the ACT1–TRAF2/5 interactions and its physiological role remain elusive.

### Hsp90

Hsp90 is a molecular chaperone protein essential for activating many signaling proteins in the eukaryotic cell (110). It has been observed that Hsp90 interacts with ACT1, but does not with the D19A loss-of-function mutation variant of ACT1 (104). In addition, Hsp90 inhibitors abolish the interaction of Hsp90 or TRAF proteins, and IL-17 signaling. Consequently, the activity of Hsp90 is required for the IL-17 signaling, and the interaction between Hsp90 and ACT1 N-terminus is critical for TRAF6-dependent IL-17-mediated response in epithelial cells (Figure 6).

### Other E3 Ligases: Peli and SCF^β*-*TrCP^

Peli (Pellino) is a family of signal-responsive E3 ubiquitin ligases regulating innate immune responses by K48 and K63-linked polyubiquitination (111). The family encompasses 3 members (Peli1, 2, and 3) that are ubiquitous and interact with TRAF6, IRAK1/4, and TAK1.

Peli1 controls both the downstream and upstream TRAF6 signaling pathway. In downstream of TRAF6, Peli1 is involved in polyubiquitination of RIPK1 and subsequent activation of TAK1–IKK–NF-κB signaling in macrophages (112). The activation of Peli1 is mediated by TBK1 and IKKε in a TRIF-dependent TLR pathway (108). In upstream of TRAF6, Peli1 functions in the polyubiquitination of IRAK1 and is required for IL-1 signaling although the precise mode of action remains unclear (113). Peli1 promotes microglial TRAF6-mediated MAPK activation in EAE (114). Specifically, Peli1 mediates TRAF6-induced K63-linked polyubiquitination of c-IAP [c-inhibitor of apoptosis protein: a member of other E3 ubiquitin ligase family IAP (115)], which then ubiquitinates TRAF3 with K48 linkage, resulting in TRAF3 degradation and thereby removing its suppression of the signaling for MAPK activation (Figure 6). Blockade of IRAK1–Peli1–TRAF6 signaling by TGF-β-mediated Smad6–Peli1 interaction is involved in the anti-inflammatory effects of TGF-β signaling (116).

Moreover, Peli1 is possibly involved in the development of psoriasis. Peli1 expression is enhanced in the epidermis of psoriasis lesions, and doxy-inducible *Peli1*^*tg*^ mice spontaneously develop psoriatic inflammation, which depends on Peli1 overexpression in radioresistant cells, with increased expression levels of IL-17 and IL-22 in the skin (115). In addition, imiquimod-induced psoriatic dermatitis is impaired in Peli1 deficient mice. These results suggest possible regulatory roles of Peli1 in IL-17 signaling in epithelial cells. In these mice, however, the involvement of Peli1 in keratinocyte-specific TRAF6 signaling remains unexplored (115).

Peli2 and Peli1 have redundant E3 ligase activities with TRAF6 in IL-1, TLR, and RANKL signaling (117). The IL-1β-induced formation of K63-polyubiquitin chains and ubiquitylation of IRAK1, IRAK4, and MyD88 are abolished in TRAF6/Peli1/Peli2 triple-knockout (KO) cells, but not in TRAF6 KO or Peli1/2 double-KO cells. In E3 ligase-inactive TRAF6 (L74H) mutant MEFs, TLR responses are reduced in the early phase but abolished in the late phase whereas RANKL signaling is unaffected. Thus, we may suggest that TRAF6 poses E3 ligase activity-dependent and independent roles.

Peli3 negatively regulates TLR3 signaling via polyubiquitination of TRAF6 as poly(I:C)-induced polyubiquitination of TRAF6 is defective in MEFs lacking Peli3, resulting in enhanced TLR3-mediated production of type I IFNs (118) and suggesting possible regulatory roles of Peli in TRAF6 signaling in epithelial cells.

Skp1-cullin-1-F-box (SCF) that contains the F-box protein β-transducin repeat-containing protein (SCF^β−*TrCP*^) is an E3 ubiquitin ligase complex. It was demonstrated that SCF^β−*TrCP*^ is involved in the desensitization of IL-17 signaling though ACT1 polyubiquitination and degradation (119). Persistent stimulation with IL-17 in HeLa cells stimulates ACT1 phosphorylation and subsequent K48-linked polyubiquitination and degradation through SCF^β−*TrCP*^, resulting in the IL-17 desensitization. However, similar regulatory mechanisms remain unknown in epithelial cells.

### Deubiquitinases: A20, CYLD, and USP25

A20 is a ubiquitin editing enzyme and is a negative regulator of innate immune responses. Single nucleotide polymorphisms (SNPs) in *TNFAIP3* encoding A20 confer risk to several inflammatory or autoimmune diseases, such as psoriasis, Crohn's disease, rheumatoid arthritis, and systemic lupus erythematosus (120, 121). A20 regulates polyubiquitination via its dual roles (Figure 6): deubiquitinating enzyme activity removing K63-linked polyubiquitin chains resulting in reduction of ubiquitination signaling; and ubiquitin E3 ligase activity that promotes K48-linked polyubiquitination and subsequent proteasome-mediated degradation of the substrate signaling molecules (121). In MEFs, A20 is associated with TRAF6 in an IL-17-dependent manner and restricts the IL-17-dependent activation of NF-κB and MAPKs (122). It has also been suggested that TNF–A20 signaling axis is responsible for TNF-mediated IL-17 inhibition in CD4+ T cells, which is related to disease exacerbation in inflammatory bowel diseases and multiple sclerosis in addition to paradoxical reactions in psoriasis as a response to anti-TNF therapies (123). Mechanistically, A20 ovarian tumor (OTU) domain at the N-terminus, which has a deubiquitinase activity, binds to TRAF6 and dismantles K63-linked polyubiquitin chains from TRAF6 (124). However, it is not followed by A20-mediated K48-linked polyubiquitination and subsequent degradation of TRAF6. In addition, the deubiquitinase activity of A20 is dispensable for NF-κB signaling in macrophages; as loss of deubiquitinase function mutation of A20 (C103A) does not affect ubiquitination and K63-linked ubiquitination levels in TRAF6 (125). Therefore, epithelial TRAF6 may not be a major target of A20 in regulating type 17 immune responses and the precise mechanisms of the cell-specific roles of A20 and their controls remain to be addressed.

*TNIP1*, encoding A20 binding and inhibitor of NF-κB-1 (ABIN1), is also associated with susceptibility to psoriasis (120). ABIN1 directly binds to A20 and NEMO/IKKγ and negatively restricts TNF and TLR-induced signals (126) (Figure 6). Loss of ABIN1 in keratinocytes (*K14-Cre Tnip1* ^*flox*/*flox*^) leads to deregulation of IL-17-induced gene expression and exaggerated chemokine production *in vitro* and overt psoriasis-like inflammation *in vivo* (127). In contrast, ABIN1 lentiviral overexpression inhibits the expression of genes for IL-17 and TNF signaling pathways in human keratinocytes *in vitro* (128). Thus, epithelial homeostasis and dysregulation of the polyubiquitination system is critical for the IL-17-mediated chronic inflammation such as psoriasis.

CYLD is another deubiquitinase that removes K63 and Met1 (M1)-linked polyubiquitin chains from several signaling mediators and thus dampens NF-κB-dependent gene expression (129) (Figure 6). CYLD has been demonstrated to negatively regulate TRAF6-mediated ubiquitination (130, 131). CYLD is required for down-regulation of RANKL signaling in osteoclasts by inhibiting TRAF6 ubiquitination (132). However, despite its significant role in modulating tumor development (including cylindroma) (133), contribution of epithelial CYLD in regulating innate and type 17 immune responses needs to be further investigated.

Of note, both A20 and CYLD deubiquitinases are cleaved by MALT1 that is activated by an upstream component of TRAF6 signaling: a CBM signalosome (134, 135) (Figure 6). Inactivation of MALT1 protease activity causes reduced stimulation-induced T cell proliferation, impaired IL-2 and TNF production, as well as defective T_H_17 differentiation *in vitro* (136). Consequently, the development of T_H_17-dependent EAE is attenuated in MALT1 protease activity-deficient mice despite their development of a multiorgan inflammatory pathology characterized by T_H_1 and T_H_2/0 responses (136). The administration of a MALT1 protease inhibitor mepazine also attenuates EAE (137). Possible contribution of CARD14 in cleaving these deubiquitinases is discussed in the first section of the next chapter.

Ubiquitin-specific protease 25 (USP25) is a newly identified deubiquitinase that negatively regulates IL-17-triggered signaling (138). IL-17 induces the association of USP25 with TRAF5 and TRAF6, and USP25 removes K63-linked ubiquitination in TRAF5 and TRAF6. USP25 deficiency enhances the expression of inflammatory mediators in lung epithelial cells and MEFs. Consistently, *Usp25*^−/−^mice show greater sensitivity to IL-17-induced pulmonary inflammation and EAE (138).

## Players in Epithelial TRAF6 Pathways in Psoriasis

### CARD14

Being a member of CARD family protein, CARD14 can bind with BCL10, MALT1, and TRAF proteins including TRAF6 (139). Also, it is involved in activation of innate immune responses by the formation of a CBM signalosome with subsequent activation of NF-κB and MAPK pathways (140, 141). CARD14 is known to be selectively expressed in the epidermis, and its gain-of-function mutations are found in the familial type of psoriasis (142). However, the receptor signaling pathways upstream of CARD14 in keratinocytes remain unspecified, yet the keratinocyte treatment with CARD14 siRNA reduces the MALT1 protease activity (143). In addition, CARD14 is involved in IL-17 pathways in keratinocytes (45). Pathogenic CARD14 mutants (such as CARD14 E138A or ΔE138) result in spontaneous formation of signalosome assembly in keratinocytes *in vitro* and development of psoriatic dermatitis *in vivo* (45, 144). IL-17 stimulates CARD14 interaction with TRAF6 and ACT1 in keratinocyte cell line HaCaT while IL-17 induces lower *Ccl20, S100a8*, and *S100a9* expression in CARD14-deficient mouse keratinocytes compared to wild-type cells *in vitro* (45).

Moreover, it has been demonstrated that the CARD14 E138A mutant activates MALT1 protease activity (145). Some pathogenic CARD14 mutants are all more potent than wild-type CARD14 in inducing A20 and CYLD cleavage but others are not (140). These results may tempt us to consider that defective regulation by A20 and CYLD for the IL-17–TRAF6-mediated responses is central for the development of psoriasis in patients with CARD14 pathogenic mutations. Consequently, CARD14 has definitive and multiple roles in the cascade/loop of IL-17–TRAF6-mediated chronic inflammation in the skin of psoriasis patients (Figure 3A).

It is of interest to note that loss-of-function mutations in *CARD14* have been reported in 3 families with a severe variant of atopic dermatitis (146) whereas *Card14*^−/−^ mice do not have spontaneous AD (45).

### NF-κB Pathways vs. MAPK Pathways

IKK–NF-κB and p38/JNK–AP-1 pathways pose major impacts on TRAF6 downstream. Both NF-κB and p38 MAPK are activated in the epidermis of the lesional skin from psoriasis patients (147–149). Therefore, it seems confusing that both NF-κB and AP-1 deficiency in the epidermis result in spontaneous development of psoriatic inflammation in mice (150–152). However, the former is a very quick outcome after birth whereas the latter shows more chronic changes suggesting a secondary outcome. In addition to the AP-1-mediated gene transcription, p38 regulates the expression of inflammatory cytokines and chemokines via their mRNA stabilization and translation (74, 75), suggesting that the phenotype of mouse epidermal AP-1 deficiency does not fully represent that of p38 deficiency. Therefore, the unbalanced homeostasis of the TRAF6 signaling pathways with attenuated NF-κB activation and dominant MAPK activation in the epidermis might contribute to triggering type 17 innate response and giving rise to the increased susceptibility to psoriasis.

**Figure 7:**
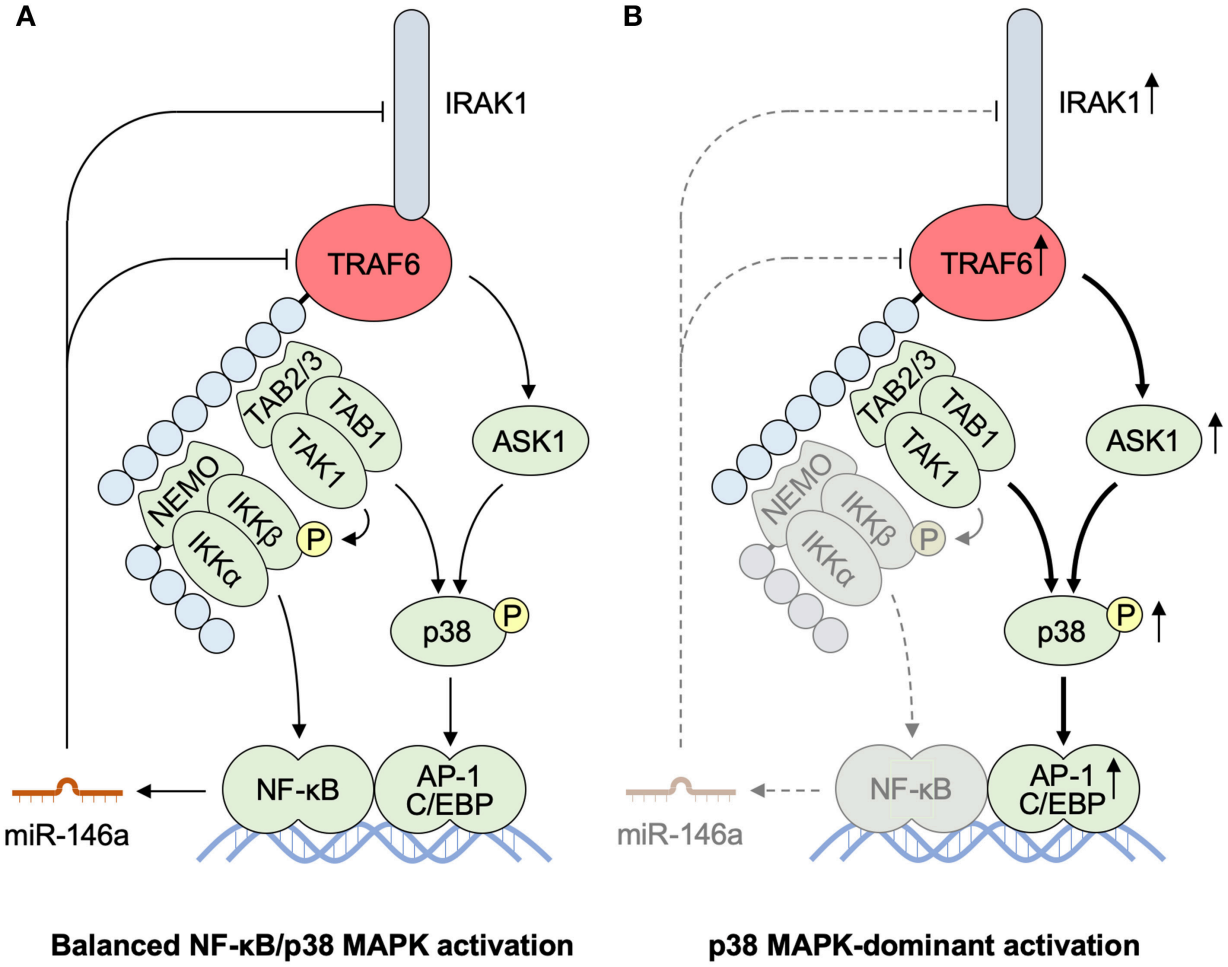
MicroRNA-mediated TRAF6 regulation balancing NF-κB and MAPK activities. **(A)** miR-146a is produced in an NF-κB-dependent manner. miR-146a interferes the transcription and translation of IRAK1 and TRAF6 as a mediator of the negative feedback pathway for an NF-κB pathway. **(B)** Defective activation of an IKK–NF-κB pathway results in the reduced production of miR-146a. The impaired miR-mediated regulation of IRAK1 and TRAF6 accelerates further activation of a p38 MAPK pathway, resulting in p38 MAPK-dominant activation. AP-1, activator protein 1; ASK1, apoptosis signal-regulating kinase 1; C/EBP, CCAAT/enhancer binding protein; IKK, IκB kinase; IRAK, interleukin-1 receptor-associated kinase; MALT1, mucosa associated lymphoid tissue lymphoma translocation gene 1; miR, microRNA; NEMO, NF-κB essential modulator; NF-κB, nuclear factor κB; TAB, TAK1 binding protein; TAK1, transforming growth factor-β-activated kinase 1; TRAF6, tumor necrosis factor receptor associated factor 6.

As for the balance between the NF-κB and MAPK activation, it is noteworthy that microRNA (miR)-146a is expressed in a NF-κB dependent manner and inhibits the transcription of TRAF6 and IRAK1, leading to negative feedback regulation of the TRAF6–NF-κB pathway (153, 154). Accordingly, miR-146a deficiency leads to hyperexpression of TRAF6 and IRAK1. Therefore, it is plausible to assume that the defective NF-κB activation in response to TLR/IL-1 or IL-17 signaling may initiate dominant activation of p38/JNK MAPKs (Figure 7). At present, however, it has not yet been determined whether cutaneous activation of p38/JNK MAPKs is sufficient for the induction of IL-17-dependent psoriatic inflammation.

### IL-36 Cytokines

Many IL-1 family cytokines, including IL-1α/β, IL-18, IL-33, and IL-36α/β/γ, share their molecular structure and protein maturation mechanism. The ligation to their functional receptors signals through MyD88-dependent TRAF6 signaling pathways (155) (Figure 2A). Imiquimod-induced skin inflammation is partially reduced in mice deficient for both IL-1α/IL-1β or for IL-1R1, but not in IL-1α- or IL-1β-deficient mice, demonstrating the redundant activity of IL-1α and IL-1β for skin inflammation (156). Limited clinical efficacy of anti-IL-1 or IL-1R antibodies in psoriasis also suggest possible redundancy of the IL-1 family (157).

Ligation of IL-36α/β/γ, but not the endogenous IL-36R antagonist (IL-36Ra), to IL-36R, activates NF-κB, and p38 MAPK pathways (158) (Figure 3B). Loss-of-function mutations in *IL36RN* encoding IL-36Ra are found in familial-type generalized pustular psoriasis (159). IL-36R-deficient (*Il36r*^−/−^) mice are protected from the imiquimod-induced expansion of dermal IL-17-producing γδ T cells and psoriatic dermatitis, and IL-36R on radioresistant resident cells is crucial for these responses (160). In addition, RNA-seq analysis of normal human epidermal keratinocytes reveals that IL-1β and IL-36 responses in keratinocytes share MyD88-dependent gene signature (161). Therefore, IL-36–TRAF6 signaling in keratinocytes might be a critical event in this animal model and human psoriasis while the production of IL-36α/β/γ is not so affected in imiquimod-induced dermatitis in mice lacking TRAF6 in keratinocytes (3). Intriguingly, IL-36 also induces IκBζ expression in keratinocytes in a MyD88-dependent manner and is required for the expression of various psoriasis-related genes (162) as described in the next section.

### IκBζ

IκBζ is the inducible nuclear protein that functions as a regulator of IL-1/TLR-mediated gene expression, such as *Il6, Il12b*, and *Csf2*, but not *Tnf* (163). *NFKBIZ* encoding IκBζ resides in a psoriasis susceptible locus (164). IL-17 induces IκBζ expression in keratinocytes in a p38 MAPK-dependent manner (165, 166) while TNF and IL-17-mediated synergistic induction of *DEFB4*, but not *CCL20* or *IL8* expression, depends on IκBζ in human keratinocytes (166) although *CCL20* and *IL8* are also IκBζ target genes (162, 167). In addition, IκBζ is directly recruited to the promoter regions of psoriasis-associated target genes (167) whereas the loss of IκBζ expression alters H3K4 tri-methylation and switch/sucrose non-fermenting (SWI/SNF) complex recruitment, thereby influencing promoter accessibility at IκBζ target genes (168, 169). Moreover, imiquimod-induced psoriatic dermatitis is fully abolished in IκBζ-deficient mice (167) whereas intradermal injection of IL-36α induces psoriatic dermatitis that is dependent on IκBζ (162). Furthermore, dysregulation of IκBζ function might be involved in the chronicity of IL-17-mediated inflammation because it has been shown that IκBζ has opposite regulatory roles at initial and resolution phases of inflammation via the DNA methylation by Tet2 (170). Consistently, dimethyl itaconate can selectively regulate secondary, but not primary, transcriptional responses to TLR stimulation via inhibition of IκBζ protein induction by ATF3 while dimethyl itaconate ameliorates IL-17–IκBζ-driven skin pathology in a mouse model of psoriasis (171). Therefore, IL-36R–TRAF6–IκBζ-IL-36 and IL-17R–TRAF6–IL-17 loops might be key features of the chronic inflammation in the EIME of psoriatic dermatitis (Figures 3A,B). Of note, IκBζ-deficient mice spontaneously develop atopic dermatitis-like inflammation with increased levels of serum IgE (172).

### RIPKs

A RIPK family is composed of 7 kinases characterized by their roles in balancing inflammation and cell death in terms of canonical and non-canonical NF-κB, MAPK, and apoptotic and non-apoptotic cell death pathways (44, 173). Despite a characteristic capacity of RIPK family members to bind to TRAF proteins, RIPK2 and RIPK4, but not RIPK1, interact with TRAF6 and get involved in TRAF6-mediated NF-κB and MAPK activation (174–177). Only a few reports have suggested a link between psoriasis and keratinocyte RIPK4 (178, 179) whereas RIPK2 might be involved in gut mucosal innate responses (31).

Among RIPKs, only RIPK2 (also known as CARD3) has a CARD domain at the C terminus (173). TLR4-induced activation of NF-κB and p38 MAPK impaired in mouse macrophages lacking RIPK2 and the kinase activity of RIPK2 is dispensable in these signaling pathways (180). RIPK2 is known to function in NLR signaling via CARD–CARD homotypic interactions between NOD1/2 and RIPK2 (173). NOD2 overexpression-induced activation of TRAF6–NF-κB signaling is inhibited by RIPK2 siRNA. NOD2 and RIPK2 share a common E2 complex to ubiquitinate NEMO/IKKγ and activate NF-κB in MEFs and human intestinal microvascular endothelial cells (31, 181). The activation of TRAF6 is lost with major Crohn's disease-associated NOD2 allele L1007insC, suggesting involvement of RIPK2 in the linkage between the mucosal innate responses and the gut microbiota (31).

RIPK4 tips toward NF-κB signaling for inflammation especially in skin cells. RIPK4 has been shown to regulate epidermal differentiation and cutaneous inflammation. Mice with epidermis-specific expression of RIPK4 (*K14-Ripk4*^*tg*^) are specifically sensitive to phorbol-12-myristate-13-acetate (PMA)-driven TNF-independent inflammation (182). Consistently, the PMA-induced expression of proinflammatory mediators is inhibited by RIPK4 siRNA treatment in human keratinocytes (183). RIPK4 expression levels are higher in keratinocytes in psoriasis lesions than in healthy control skin, and stimulation with IL-17 induces RIPK4 expression in keratinocytes (178, 179). In addition, RIPK4 interacts with STAT3 and enhances IL-17-mediated STAT3 phosphorylation and CCL20 expression in HaCaT cells. RIPK4 mutations are associated with popliteal pterygium syndrome (Bartsocas-Papas type) showing limb and skin abnormalities (184), which is considered to be a close resembling of IKKα deficiency (185, 186). Consistently, RIPK4 associates with IKKα and IKKβ and activates them in a kinase-dependent manner (187). Keratinocyte-specific ablation of RIPK4 (*K14-Cre Ripk4*^*tm*1*c*/*tm*1*c*^) also results in delayed keratinization and stratum corneum maturation (188). Either RIPK4 or IKKα down-regulation in primary keratinocytes interferes with expression of *Ovol1* (189, 190). However, the involvement of TRAF6 in RIPK4 functions in keratinocytes remains largely unknown.

## Epithelial TRAF6 Signaling in the EIME of Type 17 Responses

The epithelial tissues organize the microenvironment for the induction and propagation of situation-specific inflammation in the adjacent tissues beneath the epithelium (1, 2). This microenvironment is composed of 5 factors. Four out of them are unique in the epithelial microenvironment: (1) microbiota, (2) barriers, (3) epithelial cells, and (4) sensory nerve endings; while the 5th (immune cell society) completes the EIME. Interaction of these factors in the EIME governs the protective and regenerative responses of the epithelium. Transcriptional regulation of the epithelial cells has a central role in the organization of the EIME because both the primary responses to external agents and the secondary responses to the immune activation of epithelial cells produce inflammatory mediators essential for the amplification and propagation of effective immune responses. Accordingly, the dysregulated activation of the EIME can lead to the development of chronic inflammatory diseases in the skin, the gut, and the lung.

TRAF6 is considered to be a central factor that drives the transcriptional responses of epithelial cells in triggering and propagating type 17 immune responses, in host defense and inflammatory diseases. TRAF6 plays critical roles in the primary responses of epithelial cells to external agents that induce type 17 innate and immune responses. In addition, the epithelial cells have IL-17R while TRAF6 has an inevitable role in the IL-17 signaling. Moreover, the activation of TRAF6 signaling and downstream of NF-κB and MAPK pathways effectively promotes the transcription of proinflammatory mediators that mediate the activation of the IL-23/IL-17 axis and drive the inflammatory loop of IL-17 in the EIME (Figure 3A). Furthermore, TRAF6 is expected to be essential for driving the inflammatory loop of IL-36–IκBζ that plays indispensable roles in organizing the type 17 EIME in the skin for the development psoriatic inflammation (Figure 3B). Consequently, several players contribute to the harmonious regulation of TRAF6 signaling in the epithelial cells in order to orchestrate the architecture of type 17 innate and immune responses as described in this review.

TRAF6-dependent signaling in keratinocytes does not seem to play critical roles in T_H_1 or T_H_2-type inflammation (3). K5-*Cre Traf6* ^*flox*/*flox*^ mice develop hapten-induced T_H_1-type contact hypersensitivity as well as wild-type mice despite a significant but partial attenuation of *Ifng* expression in the skin. In addition, expression of *Il4* mRNA and serum IgE levels in papain-induced skin inflammation are comparable between K5-*Cre Traf6* ^*flox*/*flox*^ mice and wild-type mice. To our knowledge, however, there have been no additional information about the roles of epithelial TRAF6 in T_H_1, T_H_2, or Treg response, or counterpart molecules in epithelial signaling pathways for the induction of each immune responses.

## Concluding Remarks

The multilateral studies into the molecular functions and their regulatory mechanisms of TRAF6 have depicted various aspects of TRAF6 definitive roles in the immune system and inflammatory diseases. The new insights on TRAF6 signaling in epithelial cells during different immune responses provide us with the evidence for other potential roles rather than serving as barriers. One may expand the idea to the correspondence of specific cell signaling in the epithelial cells to certain type of immune response or chronic inflammation. However, these insights may raise more questions than answers: (1) Is epithelial TRAF6 signaling also essential for type-17 protective immune responses against microbes, such as Candida or segmented filamentous bacteria? (2) Is epithelial TRAF6 critical in the protective responses and inflammatory diseases in other epithelial organs such as the respiratory system or the urinary tract? (3) Does epithelial TRAF6 have definitive roles in T_H_1, T_H_2, or Treg response or differentiation in some situations? (4) Otherwise, what is the epithelial counterpart signaling molecule in type 1, type 2, or regulatory immune responses? (5) What “balance” of the downstream effectors can represent the decision on the consequent immune types? (6) What are the mechanisms responsible for the community of the EIME loop of inflammation in chronic inflammatory diseases?

The progress in our understandings of the regulation of immune responses by TRAF6 in the epithelial cells will arrow us to develop new therapeutic strategies. CD40–TRAF6-specific nanobiologics have been demonstrated to be effective *in vivo* (191, 192). These preceding investigations should enhance developing the new drugs that can modulate TRAF6 E3 ubiquitinase activities or protein–protein interactions with TRAF6 for specific purposes such as effective cutaneous vaccinations and treating chronic inflammatory diseases.

## Author Contributions

All authors listed have made a substantial, direct and intellectual contribution to the work, and approved it for publication.

### Conflict of Interest Statement

The authors declare that the research was conducted in the absence of any commercial or financial relationships that could be construed as a potential conflict of interest.
